# Impact of surface receptors TLR2, CR3, and FcγRIII on *Rhodococcus equi* phagocytosis and intracellular survival in macrophages

**DOI:** 10.1128/iai.00383-23

**Published:** 2023-11-29

**Authors:** Bibiana Petri da Silveira, Rola Barhoumi, Jocelyne M. Bray, Hannah M. Cole-Pfeiffer, Cory J. Mabry, Robert C. Burghardt, Noah D. Cohen, Angela I. Bordin

**Affiliations:** 1 Department of Large Animal Clinical Sciences, Equine Infectious Disease Laboratory, Texas A&M University, School of Veterinary Medicine & Biomedical Sciences, College Station, Texas, USA; 2 Department of Veterinary Integrative Biosciences, Texas A&M University, School of Veterinary Medicine & Biomedical Sciences, College Station, Texas, USA; Universite de Geneve, Geneva, Switzerland

**Keywords:** bacterial infection, complement receptor, macrophages, phagocytosis, pneumonia, toll-like receptor, pathogen-recognition receptor

## Abstract

The virulence-associated protein A (VapA) produced by virulent *Rhodococcus equi* allows it to replicate in macrophages and cause pneumonia in foals. It is unknown how VapA interacts with mammalian cell receptors, but intracellular replication of avirulent *R. equi* lacking *vapA* can be restored by supplementation with recombinant VapA (rVapA). Our objectives were to determine whether the absence of the surface receptors Toll-like receptor 2 (TLR2), complement receptor 3 (CR3), or Fc gamma receptor III (FcγRIII) impacts *R. equi* phagocytosis and intracellular replication in macrophages, and whether rVapA restoration of virulence in *R. equi* is dependent upon these receptors. Wild-type (WT) murine macrophages with TLR2, CR3, or FcγRIII blocked or knocked out (KO) were infected with virulent or avirulent *R. equi*, with or without rVapA supplementation. Quantitative bacterial culture and immunofluorescence imaging were performed. Phagocytosis of *R. equi* was not affected by blockade or KO of TLR2 or CR3. Intracellular replication of virulent *R. equi* was not affected by TLR2, CR3, or FcγRIII blockade or KO; however, avirulent *R. equi* replicated in TLR2^-/-^ and CR3^-/-^ macrophages but not in WT and FcγRIII^-/-^. rVapA supplementation did not affect avirulent *R. equi* phagocytosis but promoted intracellular replication in WT and all KO cells. By demonstrating that TLR2 and CR3 limit replication of avirulent but not virulent *R. equi* and that VapA-mediated virulence is independent of TLR2, CR3, or FcγRIII, our study provides novel insights into the role of these specific surface receptors in determining the entry and intracellular fate of *R. equi*.

## INTRODUCTION

*Rhodococcus equi* is a facultative intracellular zoonotic pathogen mostly recognized for causing severe pneumonia in foals and immunocompromised people (1–4). *R. equi* virulence in foals is attributed to a plasmid of approximately 80–85 kilobase-pairs known as pVAPA, which contains a pathogenicity island that encodes a family of *vap* genes, including the virulence-associated protein A (VapA) (5–9). VapA confers on *R. equi* the ability to replicate inside macrophages, and strains lacking pVAPA do not survive intracellularly and are avirulent in foals (5, 8, 9). The mechanisms by which VapA promotes virulence are still being investigated, but it is hypothesized that VapA acts by permeabilizing phagosomal and lysosomal membranes, generating a pH-neutral and growth-promoting intracellular niche in macrophages (10). It has been proposed that VapA is necessary but not sufficient for virulence (11); however, the addition of recombinant VapA (rVapA) has been previously shown to rescue intracellular replication in avirulent plasmid-cured *R. equi* (10, 12, 13). During *in vitro* intracellular infection, rVapA interacts with the phosphatic acid of liposomes in the host cell membranes (13), but it is unknown how rVapA interacts with specific mammalian cell receptors.

Previous studies have suggested that Toll-like receptor 2 (TLR2), complement receptor 3 (CR3, CD11b/CD18, or Mac-1), and Fc gamma receptors (FcγRs) have important roles in *R. equi* infection, such as VapA binding, phagocytosis, and bacterial intracellular fate (14–16). TLR2 recognizes pathogen-associated molecular patterns present in *R. equi*, such as lipoteichoic acid, lipoarabinomannan, among others (17–19), and is activated by infection with *R. equi* or rVapA alone, inducing pro-inflammatory cytokine expression in macrophages (15). There is conflicting evidence about the importance of TLR2 for killing *R. equi* in mice: TLR2^-/-^ mice failed to clear virulent *R. equi* infection (15), whereas TLR2^-/-^ mice that received a VapA vaccine were protected against *R. equi* infection (20). This vaccine, however, contained an attenuated Salmonella expressing VapA, and other factors such as non-specific stimulation of the innate immune system (21) could play a role in protection. Therefore, a better understanding of the role of TLR2 activation in *R. equi* intracellular fate in macrophages is needed.

TLR2 has been shown to regulate CR3 activation (22–24). Interaction of bacterial surface components with TLR2 enhances the ligand-binding capacity of CR3 through the activity of phosphatidylinositol 3-kinase (22–24), including increased CR3 avidity for mycobacteria (25). CR3-mediated entry has been shown to alter bacterial fate differently for different pathogens: in *Porphyromonas gingivalis* infection (both *in vitro* and *in vivo*), it promotes bacterial survival and virulence (23, 26, 27); conversely, in both *in vitro* and *in vivo* infection with virulent *Mycobacterium tuberculosis,* intracellular survival remains unaffected by CR3 (28–30). In addition, phagocytosis *via* complement receptors induces proinflammatory cytokine production (31). Despite CR3 being canonically recognized as the exclusive receptor for binding and internalizing *R. equi* by mammalian cells (14), the role of CR3-mediated phagocytosis in the fate of intracellular *R. equi* is ill-defined. Unlike TLR2, no studies on CR3^-/-^ mice or cell lines were conducted to specifically determine the effects of the absence of this receptor during *R. equi* infection.

Receptors selective for the Fc region of immunoglobulin G (IgG) can have distinct stimulatory (FcγRI, FcγRIII, and FcγRIV) or inhibitory (FcγRII) effects on inflammatory cells (32). Interaction between FcγRs on monocytes and macrophages and their ligands stimulates antimicrobial activities such as cytokine synthesis (33), respiratory burst (34), and intracellular bacteria killing (35). When intracellular pathogens are internalized by FcγRs, they are killed more efficiently by phagocytes (16). In *Pseudomonas aeruginosa* infection*,* FcγRIII expression confers protection against pneumonia, although phagocytosis was not quantitatively different between WT and FcγRIII^-/-^ murine macrophages (36). In *R. equi* infection, however, the role of FcγRs has not been specifically investigated and no studies using either a cell line or mice FcγRIII^-/-^ were performed. Moreover, studies have focused on receptor ligands (IgG from serum/plasma) rather than on the receptors on the surface of phagocytes *per se* (37–40). *R. equi* opsonization with serum or plasma increases phagocytosis and bacterial killing by phagocytes, but these effects might be attributable to complement, antibodies, or other serum components (14, 37–40). Protection against *R. equi* pneumonia induced by specific hyperimmune plasma (HIP) transfusion in newborns has been attributed primarily to the presence of *R. equi*-specific antibodies, in particular IgG (41–46). *In vitro* opsonization with either HIP or standard plasma (from donors not hyperimmunized against *R. equi*) decreased intracellular survival of *R. equi*, but the mechanisms by which standard plasma or HIP protect against *in vitro* infection are poorly understood (39, 41).

Despite the supposed role of TLR2, CR3, and FcγRIII surface receptors in the interaction between *R. equi* and macrophages, the direct impact of these receptors has not been addressed, and how the absence of specific surface receptors affects *R. equi* phagocytosis and killing by macrophages remains unknown. The objectives of our study were to determine the roles of TLR2, CR3, or FcγRIII on phagocytosis and intracellular replication of *R. equi* in murine macrophages, and to determine whether rVapA restoration of intracellular replication in avirulent *R. equi* is dependent upon these receptors. To our knowledge, this is the first report comparing the effects of knocking out TLR2, CR3, and FcγRIII in macrophage phagocytosis and killing of *R. equi*.

## MATERIALS AND METHODS

### rVapA production

The plasmid pGEX-2TK (GE Healthcare Life Sciences, Piscataway, NJ, USA) was used for the expression of rVapA as a glutathione S-transferase (GST) fusion protein. The *vapA* gene sequence was codon-optimized and the resulting sequence (Fig. S1) was inserted to create pGEX-2TK-VapA. Bacteria were grown in Luria-Bertani Broth (Miller; VWR Chemicals, Solon, OH) with 50 µg/mL of ampicillin at 130 rpm and 37°C until an optical density between 0.7 and 0.8 at 600 nm was detected (OD_600_; Genesys 20, Thermo Scientific, Waltham, MA, USA). The expression of GST-VapA fusion protein was induced by the addition of 2–4 mM of isopropyl β-D-1-thiogalactopyranoside (IPTG; Sigma-Aldrich, St. Louis, MD, USA) and rotated at 130 rpm overnight at 15°C. The bacterial cell pellet was harvested by centrifugation at 2,000× *g* (5810R, Eppendorf AG, Hamburg, Germany), disrupted with 5 mL of lysis buffer per g of pellet, and incubated for 15 min at room temperature (RT) on a rocking platform (Model 200, VWR, Radnor, PA, USA). Lysis buffer was made using 10 µg/mL of lysozyme from chicken egg white (Sigma-Aldrich, St. Louis, MO, USA) in B-PER Bacterial Protein Extraction Reagent (Thermo Scientific, Rockford, IL, USA) solution supplemented with 1× proteases inhibitor cocktail (Sigma-Aldrich, St. Louis, MD, USA). Lysate was harvested by centrifugation, clear supernatant was filtered (0.22 µm), and purified with a column using glutathione sepharose 4B beads (GE Healthcare Life Sciences, Piscataway, NJ, USA) according to the manufacturer’s instructions. Eluted protein was concentrated and buffer-exchanged [1× phosphate-buffered saline (PBS), Lonza, Walkersville, MD, USA] using SpinX20 10K MWCO membrane (Corning, Oneonta, NY, USA). VapA-GST was digested with thrombin (1 unit/100 µg of protein; GE Healthcare Life Sciences, Piscataway, NJ, USA) overnight at 4°C on a rotator (Model 13916–822, VWR, Radnor, PA, USA). The GST tag was removed by running the digested VapA/GST over a column of glutathione sepharose 4B beads (GE Healthcare Life Sciences, Piscataway, NJ, USA) and capturing rVapA in the flow-through. rVapA was concentrated to 1–5 µg/mL using a SpinX 20, 5K MWCO column (Corning, Oneonta, NY, USA). rVapA concentration was determined using a Pierce BCA Protein Assay Kit (Thermo Scientific, Rockford, IL, USA).

To confirm rVapA purity and GST removal efficiency, a western immunoblot for GST and rVapA was performed using monoclonal antibodies (mAb; Table 1). Three micrograms of VapA, VapA-GST, or GST was diluted and incubated with Laemmli sample buffer with 5% β-mercaptoethanol (Biorad, Hercules, CA, USA) at 100°C for 5 min. Prepped samples were run on a 4%–20% Mini-PROTEANR TGX Precast Gel (Biorad, Hercules, CA, USA), the gel was imaged (ChemiDoc Touch; Biorad, Hercules, CA, USA), and then proteins were transferred onto a 0.2 µm PVDF membrane using the Trans-BlotR Turbo transfer system (Biorad, Hercules, CA, USA). Thereafter, the membrane was blocked using blocking buffer 10% non-fat milk in 1× Tris-buffered saline (TBS; Corning, Oneonta, NY, USA) incubated for 1 h at RT with agitation. rVapA was detected by the primary antibody anti-VapA E-6 (Santa Cruz Biotechnology; Table 1), diluted in blocking buffer, and incubated with the membrane for 1 h. The membrane was washed 4× with 1× Tris-buffered saline with 0.1% Tween 20 detergent (TBST) rocking for 5 min each and then the membrane was incubated for 1 h with the secondary goat anti-mouse IgG-HRP (Santa Cruz Biotechnology; Table 1) in blocking buffer. On a separate membrane, GST was detected by murine anti-GST A-6 conjugated to HRP (Santa Cruz Biotechnology, Table 1) in a blocking buffer and incubated for 1 h on the rocker (Model 200, VWR, Radnor, PA, USA). Separately, both membranes were washed 4× with TBST rocking for 5 min each and developed using SuperSignal West Femto for 2 min (Thermo Scientific, Rockford, IL, USA; Fig. S2).

**TABLE 1:** Antibodies used in this study

Antibody	Target	Dilution	Application	Cat #	Manufacturer
Monoclonal Mouse IgG_2a_	VapA	1:300	WB (primary)	sc-390576	Santa Cruz Biotechnology
Goat Polyclonal IgG - HRP	Mouse IgG (anti-VapA)	1:1000	WB (secondary)	sc-2055	Santa Cruz Biotechnology
Monoclonal Mouse IgG_1_ - HRP	GST	1:300	WB (primary)	sc-374171	Santa Cruz Biotechnology
Polyclonal Goat IgG	Mouse TLR2	1:500	WB (primary)	AF1530	R&D Systems
Polyclonal Rabbit IgG	Mouse CD11b (CR3)	1:500	WB (primary)	NB110-89474	Novus Biologicals
Polyclonal Goat IgG	Mouse FcγRIII (CD16)	1:1,000	WB (primary)	AF1960	R&D Systems
Polyclonal Rabbit - HRP	Goat IgG (anti-TLR2)	1:2,000	WB (secondary)	HAF017	R&D Systems
Polyclonal Goat IgG -HRP	Rabbit IgG (anti-CR3)	1:1,500	WB (secondary)	A0545	Sigma-Aldrich
Polyclonal Donkey IgG - HRP	Goat IgG (anti- FcγRIII)	1:4,000	WB (secondary)	705–035-147	Jackson ImmunoResearch
Monoclonal Rat IgG_2b_ (clone M1/70)	Mouse/human CD11b (CR3)	1:200	Image (primary) and blockade	101231	BioLegend
Monoclonal Rabbit IgG_k_	Mouse CD16/32 (FcγRIII)	1:200	Image (primary) and blockade	NBP2-52644-0	Novus Biologicals
Polyclonal Goat IgG – AF555	Rat IgG (CR3)	1:1,000	Image (secondary)	A21443	Invitrogen
Polyclonal Goat IgG – AF647	Rabbit IgG (FcγRIII)	1:1,000	Image (secondary)	A21244	Invitrogen
Monoclonal Mouse IgG1 (clone T2.5)	Mouse TLR2	1:200	Blockade	Mab-mtlr2	InvivoGen
Monoclonal Rat IgG2b FITC	Mouse TLR2	1:400	Flow cytometry	11–9021-80	Invitrogen

Prior to rVapA supplementation, possible endotoxin contamination was removed using Pierce High-Capacity Endotoxin Removal Spin Columns, 0.5 mL (Thermo Scientific, Rockford, IL, USA) according to the manufacturer’s instructions. Next, residual endotoxin contamination was measured to be 0.06 EU/mL for 500 nM rVapA using ToxinSensor Chromogenic LAL Endotoxin Assay Kit (Genscript, Piscataway, NJ, USA). The final rVapA concentration was determined to be 1 µg/mL using Pierce BCA Protein Assay Kit (Thermo Scientific, Rockford, IL).

### CRISPR/Cas9 sgRNA transfection for generation of TLR2^-/-^, CR3^-/-^, or FcγRIII^-/-^ J774A.1 macrophage

J774A.1 murine macrophages were obtained from the American Type Culture Collection (ATCC; Manassas, VA), and TLR2^-/-^, CR3^-/-^, and FcγRIII^-/-^ were generated in our laboratory. Prior to transfection, cells were harvested and washed in 1× PBS (Lonza, Walkersville, MD, USA). Each reaction was incubated with 100 picomoles of sgRNA for TLR2 and FcγRIII or 75 picomoles for CR3 receptor (Table 2) (Synthego, Redwood City, CA, USA), 20 pmol Cas9 (Synthego, Redwood City, CA, USA), and resuspended in buffer R (Invitrogen, Waltham, MA, USA). After a 15-min incubation at RT, 1 µg pJTI R4 Exp CMV EmGFP pA Vector (Life Technologies Corporation, Carlsbad, CA, USA) was added to each reaction along with 1 × 10^5^ cells. Cells were transfected using 1 pulse of 20 ms at 1,660 volts (Neon Transfection System, Invitrogen, Waltham, MA, USA). Cells were then placed in 1 mL of pre-warmed Dulbecco’s modified Eagle medium (DMEM; Lonza, Walkersville, MD, USA) with 10% heat-inactivated fetal bovine serum (HI-FBS; Sigma-Aldrich, St. Louis, MD, USA), 1% 100 × non-essential amino acids (NEAA; Lonza, Walkersville, MD, USA), 1% 100 × Glutamax (Gibco, Waltham, MA, USA), 100 IU/mL penicillin-G and 100 µg/mL streptomycin (Gibco, Waltham, MA, USA). After 3 days of incubation at 37°C 5% CO_2_, green fluorescent protein-positive (GFP^+^) cells were sorted individually using a Beckman Coulter Moflo Astrios Cell Sorter. Following sorting, GFP^+^ cells were expanded for downstream applications.

**TABLE 2:** Oligonucleotide sequences used to generate and confirm TLR2, CR3, and FcγRIII knockouts

Purpose	Target	Sequence of oligonucleotides 5′-3′	Amplicon size
sgRNA TLR2	TLR2	UUGGCUCUUCUGGAUCUUGG	
sgRNA CR3	CR3	UGAAGCCAUGACACAAGGCU	
sgRNA FcγRIII	FcγRIII	UUUGUAGUGAUGAUACCUCA	
Forward primer for PCR	TLR2	CCGAACCAGGAGGAAGATAAAC	486 bp
Reverse primer for PCR	TLR2	ATGAGGGAGAGAGACAGAAAGA	
Forward primer for PCR	CR3	GCTCCCCTGAGGACTTTACC	427 bp
Reverse primer for PCR	CR3	GATGGATTTCTGTGTGGTGGC	
Forward primer for PCR	FcγRIII	GCCAGGTCCAATCCAGCTAC	415 bp
Reverse primer for PCR	FcγRIII	GGAGCCTGGTGCTTTCTGAT	
Forward primer for Sanger sequencing	TLR2	GTTCAAGACTGCCCAGAGAATA	

Generation of TLR2, CR3, and FcγRIII knockouts was confirmed by PCR and Sanger sequencing. TLR2^-/-^, CR3^-/-^, and FcγRIII^-/-^ clones were harvested individually and gDNA was isolated using NucleoSpin DNA RapidLyse (Macherey-Nagel, Düren, Germany). PCR was conducted with a Phusion High-Fidelity PCR kit (New England Biolabs, Ipswich, MA, USA) according to the manufacturer’s instructions using 0.5 µM of each specific forward and reverse primer (Table 2) and 200 ng genomic DNA. Cycle conditions used were as follows: initial denaturation 98°C for 30 s, denaturation 98°C 10 s/annealing 54 (TLR2 and FcγRIII) or 59°C (CR3) 10 s/extension 72°C 20 s for a total of 40 cycles, and final extension 72°C for 5 min. PCR products were run on a 1% agarose gel and cut at each amplicon size (485 bp for TLR2, 427 bp for CR3, and 415 bp for FcγRIII). PCR products were cleaned with NucleoSpin Gel and PCR Clean-up columns (Macherey-Nagel, Düren, Germany). Purified PCR products were sequenced by Eton Biosciences (San Diego, CA, USA) using the forward primers in Table 2. TLR2^-/-^, CR3^-/-^, and FcγRIII^-/-^ sequences were compared to J774A.1 WT to confirm nucleotide deletion.

**Fig 1:**
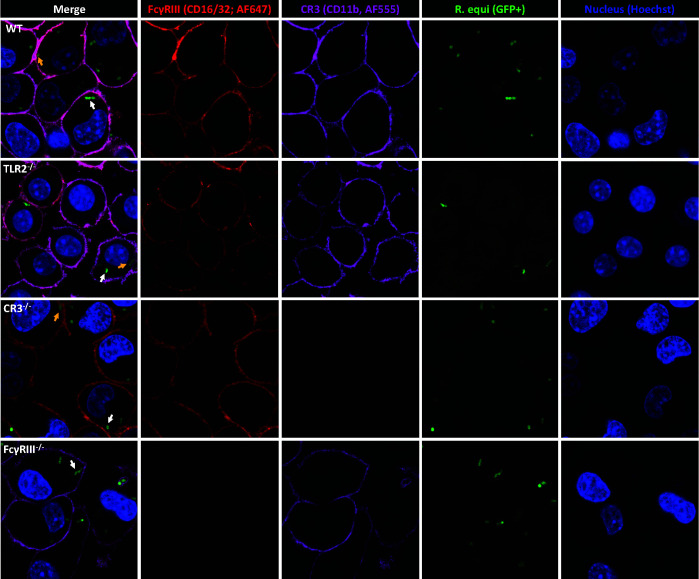
TLR2, CR3, or FcγRIII receptors are not required for *Rhodococcus equi* phagocytosis. J774A.1 WT, TLR2^-/-^, CR3^-/-^, and FcγRIII^-/-^ were infected with virulent *R. equi* (GFP^+^103^+^), and immunostaining of receptors CR3 (CD11b-AF647) and FcγRIII (CD16/32-AF555) and nuclei stained with Hoechst 33342 were performed. White arrows indicate intracellular *R. equi* and yellow arrows indicate *R. equi* adjacent to receptors.

**Fig 2:**
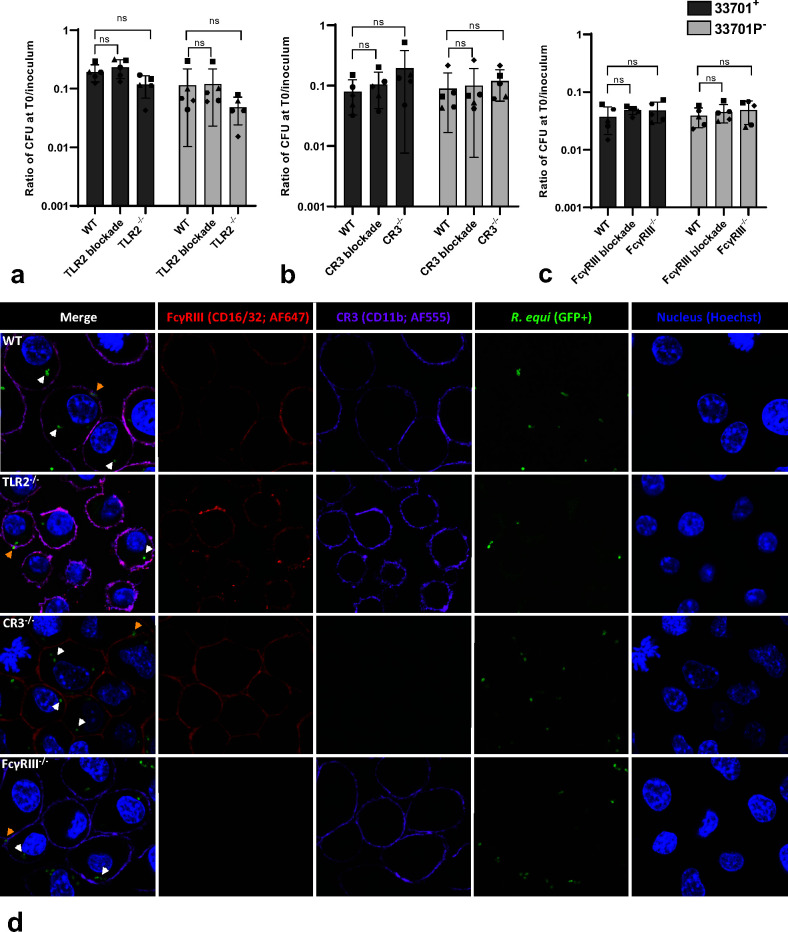
TLR2, CR3, or FcγRIII receptors are not required for *Rhodococcus equi* phagocytosis. J774A.1 murine macrophages with surface receptors (TLR2, CR3, or FcγRIII) either blocked by monoclonal antibodies (mAb) or knocked out (KO) were infected with either virulent (33701^+^) or avirulent (33701P^-^) *R. equi*. In a, b, and c, macrophages were washed, lysed, and diluted immediately (T0) for bacterial determination. Phagocytosis was calculated as a ratio of CFU at T0 divided by CFU of the bacterial inoculum, and it did not significantly (*P* < 0.05) differ between WT and mAb blocked or KO TLR2 (a), CR3 (b), or FcγRIII (c) for either virulent or avirulent *R. equi*. Five independent experiments (represented by different symbols) were performed with each experimental condition performed in triplicate. The gray bars represent the mean ratio, the error bars the standard deviation, * statistical difference (*P* < 0.05), and ns (*P* > 0.05). In d, J774A.1 WT, TLR2^-/-^, CR3^-/-^, and FcγRIII^-/-^ were infected with avirulent *R. equi* (GFP^+^103^-^). Immunostaining of receptors CR3 (CD11b-AF647) and FcγRIII (CD16/32-AF555) and nuclei stained with Hoechst 33342 were performed. White arrows indicate intracellular *R. equi* and yellow arrows indicate *R. equi* adjacent to receptors.

The absence of CR3 and FcγRIII was demonstrated by immunofluorescence (Fig. 1 and Fig. 2d) and the absence of TLR2 by flow cytometry (Fig. S3). For flow cytometry, J774A.1 WT and TLR2^-/-^ were stimulated with either 0 or 500 ng/mL of LPS for 8 h (L6529; Sigma-Aldrich, St. Louis, MD, USA), stained with anti-mouse TLR2-FITC (Table 1), and 10,000 events were acquired on a flow cytometer (Attune NxT; Invitrogen). Flow cytometry data were analyzed using FlowJo v10.7.1 Software (BD Life Sciences).

### Bacterium and inoculum preparation

*R. equi* virulent strain 33701^+^ (33701 ATCC© *Rhodococcus equi* [Magnusson] Goodfellow and Alderson with virulence plasmid expressing VapA^+^ strain; Rockville, MD, USA) and avirulent strain 33701P^-^ (virulence-plasmid-cured 33701^+^) were used for *in vitro* infection of murine macrophages. One colony of each strain was inoculated into BD brain-heart infusion broth (BHI; Becton Dickinson, Sparks, MD, USA) and shaken for 24 h at 37°C. The bacterial suspension was then centrifuged at 3,400× *g* (5810R, Eppendorf AG, Hamburg, Germany) for 10 min at 4°C. The supernatant was discarded, and the pellet was washed twice with 50 mL of PBS. The bacterial pellet was resuspended in PBS, the concentration of bacteria was adjusted spectrophotometrically (Genesys 20, Thermo Scientific, Waltham, MA USA) to an OD_600_ of 1.0 (approximately 2 × 10^8^ CFU/mL), and aliquots were stored frozen at −80°C until used. In our experience, the viability of *R. equi* after freezing to −80°C is reduced to 1/3 of the initial concentration in the first few weeks, at which point viability is maintained at a constant level for several months. To confirm the use of frozen bacteria would not affect phagocytosis and intracellular survival assays we tested the use of fresh or frozen virulent and avirulent *R. equi* stocks to infect J774A.1 murine WT macrophages as described below. Because freezing did not affect *R. equi* virulence and phenotype infecting macrophages (Fig. S4), frozen stocks were used for intracellular survival assays. Bacteria were confirmed to be virulent (*VapA* positive) or avirulent (*VapA* negative) by PCR in each bacterial stock (47). These stocks were used for intracellular survival assays.

For the imaging assays, a fresh inoculum was prepared from a single colony of GFP-expressing *R. equi* created by the transformation of strains 103^+^ and 103^-^ (*vapA* positive and *vapA* negative, respectively) (GFP^+^103^+^ and GFP^+^103^-^) with pGFPmut2 (13, 48), kindly provided by Dr. Mary Hondalus from the University of Georgia. One colony from a plate of BHI agar supplemented with 180 mg/L of hygromycin B (Sigma-Aldrich, St. Louis, MD, USA) was used to inoculate BHI broth and incubated shaken at 37°C for 24 h. The pellet was washed twice, dissolved in PBS, and OD_600_ was measured as described above. Bacterial concentration was determined spectrophotometrically to be approximately 3 × 10^8^ CFU/mL.

For all *in vitro* infections, bacteria were opsonized with 5% of non-heat-inactivated normal mouse serum or non-heat-inactivated poly-N-β-(1→6)-acetyl-glucosamine (PNAG) hyperimmune mouse serum (PHIS; kindly provided by Drs. Colette Cywes-Bentley and Gerald Pier) by incubating at 37°C for 30 min on the aliquot mixer (Ames 4651, Miles Scientific). A quantitative culture was performed to determine bacterial inoculum by plating out 10-fold dilutions in BHI agar plates and incubating at 37°C for 48 h.

### Confocal laser scanning microscopy

To evaluate phagocytosis, approximately 70,000 WT, TLR2^-/-^, CR3^-/-^, or FcγRIII^-/-^ J774A.1 cells were added to each chamber of a μ-slide 8-well coverslip (ibid, Grafelfing, Germany) and incubated overnight to adhere at 37°C 5% CO_2_. The medium was then removed and 200 µL of opsonized either virulent or avirulent *R. equi* (GFP^+^103^+^ or GFP^+^103^-^, respectively) were added at a multiplicity of infection (MOI) of 20 bacteria per macrophage into each well. The slide was then spun for 1 min at 150 g at RT, and wells were washed twice with warm PBS and fixed with 1% paraformaldehyde aqueous solution (PFA; Electron Microscopy Sciences, Hatfield, PA, USA). Rat anti-mouse CD11b and rabbit anti-mouse CD16/32 (clone 2.4G2; Table 1) mAbs were diluted in PBS, added to each well to stain CR3 and FcγRIII, respectively, and incubated at 37°C for 1 h. Wells were then washed twice with PBS and cells were incubated in the dark for 1 h with the respective secondary antibodies (Table 1). For nuclei staining, 0.5 µg/mL of Hoechst 33342 dye (Life Technologies Corporation, Carlsbad, CA, USA) was added to the wells and incubated for 10 min. The wells were washed twice with PBS and PFA 1% was added for 3D imaging in a Zeiss LSM 780 confocal microscope. Control wells included non-infected cells with receptors and nuclei stained, and infected cells were stained with secondary antibodies only. Confocal imaging was used to qualitatively determine phagocytosis. For all 3D images, four areas per well were captured.

To evaluate the effect of rVapA on lysosomal acidification of *R. equi*-infected macrophages, approximately 35,000 J774A.1 cells in a medium were added to each chamber of a μ-slide 8-well coverslip (ibidi, Grafelfing, Germany) and incubated for 2 h at 37°C 5% CO_2_. Then, 500 nM of rVapA was added to designated wells and incubated overnight using the same conditions. Cells were maintained uninfected, or infected with opsonized virulent or avirulent *R. equi* (GFP^+^103^+^ or GFP^+^103^-^, respectively) at MOI of 10 into each well and incubated for 30 min. All wells were washed twice with warm PBS and incubated for 24 h with medium supplemented with gentamycin at 8 µg/mL. The medium was removed and wells were incubated with 0.5 µg/mL of Hoechst 33342 dye (Life Technologies Corporation, Carlsbad, CA, USA) in PBS for 10 min, and then washed twice with warm PBS. Lysotracker Red DND-99 (Life Technologies Corporation, Eugene, OR, USA) was diluted to 50 nM in phenol-free DMEM (Lonza, Walkersville, MD, USA) with 10% FBS, added into each well, and incubated for 30 min. Lysotracker Red DND-99 was replaced by phenol-free DMEM with 10% FBS only and imaged in a Zeiss LSM 780 confocal microscope.

### Macrophage infection

J774A.1 murine macrophages (ATCC© J774A.1 TIB-67) were used to determine the effect of blockade or knockout of surface receptors (TLR2, CR3, or FcγRIII) on phagocytosis and intracellular survival of either virulent or avirulent *R. equi*, or avirulent *R. equi* in the presence of rVapA. Surface receptor blockade was performed by incubating cells with anti-mouse TLR2, CR3, or FcγRIII mAbs (Table 1) at 37°C and 5% CO_2_ for 30 min before and during *in vitro* infection. Macrophage infections were performed in DMEM (Lonza, Walkersville, MD, USA) with 10% HI-FBS (Sigma-Aldrich, St. Louis, MD, USA), 1% 100× NEAA (Lonza, Walkersville, MD, USA), and 1% 100× Glutamax (Gibco, Waltham, MA).

Murine macrophage phagocytosis and intracellular killing assays were performed as previously described for equine cells (49–51). Briefly, 1 mL of supplemented DMEM containing approximately 200,000 macrophages/mL was added in each well of a Costar 24-well cell culture plate (Corning Incorporated, Kennebunk, ME, USA) and incubated overnight at 37°C 5% CO_2_ to allow cells to adhere to the plate. Macrophages were then infected with opsonized *R. equi* (MOI 1:1). After 30 min of infection, cells were washed twice with PBS to remove extracellular bacteria, and then lysed immediately post-infection (T0) or after 48-h incubation in a medium supplemented with gentamycin at 8 µg/mL (T48). Cells were lysed with 1 mL of sterile water for 45 min at 37°C 5% CO_2_, scraped with a pipette tip, and vortexed and sonicated in polypropylene tubes. The number of intracellular *R. equi* was determined by quantitative culture of 10-fold dilutions in BHI agar plates. The phagocytosis ratio was calculated by dividing CFU at T0 by the inoculum, and the survival ratio was calculated by dividing CFUs at T48 by T0. Three to five independent experiments were performed with triplicate wells for each condition.

### Recombinant VapA supplementation

In wells assigned to recombinant VapA supplementation, WT or KO J774A.1 murine macrophages were seeded and incubated for 2 h then rVapA was added at 10, 100, 500, or 1,000 nM, followed by overnight incubation, and kept during *in vitro* infection.

### Statistical analysis

Data were analyzed using linear mixed-effects models with the outcome (dependent variable) of the ratios of bacterial counts of added bacteria/T0 and T48/T0 representing bacterial phagocytosis and intracellular survival, respectively. Analysis was performed using the nlme package in R software (version 3.5.1, R Foundation for Statistical Computing, Vienna, Austria), with *post hoc* pair-wise comparisons made with the method of Tukey using the multcomp package in R. Confidence intervals were estimated using maximum likelihood methods. Significance was set at *P* < 0.05.

## RESULTS

### Recombinant VapA restores intracellular replication of avirulent *R. equi*

**Fig 3:**
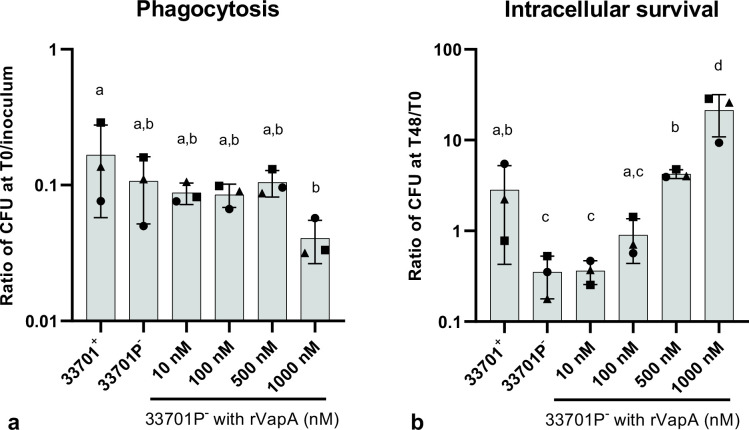
*Rhodococcus equi* phagocytosis and intracellular survival in wild-type J774A.1 supplemented with different concentrations of recombinant rVapA (rVapA). Murine macrophage monolayers were incubated overnight with 0, 10, 100, 500, or 1,000 nM rVapA and then infected with either virulent (33701^+^) or avirulent (33701P^-^) *R. equi*. Macrophages were then washed and either lysed and diluted immediately (**T0**) or cultured for 48 h and then lysed and diluted (**T48**) for bacterial determination. Phagocytosis was calculated as a ratio of CFU count at T0 divided by the CFU count of the bacterial inoculum. Intracellular survival was calculated as a ratio of CFU count at T48 divided by CFU count at T0. (**a**) No significant differences in the phagocytosis ratio between virulent and avirulent *R. equi*, or between avirulent with or without rVapA were observed. (**b**) Intracellular survival of virulent *R. equi* was significantly higher than avirulent *R. equi*, and supplementation with 500 nM of rVapA increased intracellular replication of avirulent *R. equi* similar to virulent strain. Three independent experiments (represented by symbols) were performed with each experimental condition performed in triplicate. The gray bars represent the mean ratio, the error bars the standard deviation, and different letters represent the statistical difference (*P* < 0.05) between tested conditions.

We assessed the ability of rVapA to restore the *R. equi* virulent phenotype. We first demonstrated that phagocytosis or intracellular survival of either strain of *R. equi* either frozen or freshly prepared did not significantly differ (Fig. S4), and therefore, used frozen stocks of bacteria for subsequent experiments. Phagocytosis of virulent (33701^+^) and avirulent (33701P^-^) *R. equi* by WT murine macrophages did not differ significantly (shown in Fig. 3a). In addition, overnight rVapA supplementation at 10, 100, 500, and 1,000 nM did not significantly affect phagocytosis of avirulent *R. equi*. As previously reported, intracellular survival was significantly different between virulent and avirulent *R. equi* (*P* < 0.001) (shown in Fig. 3b) (8, 12, 13, 52). Our results indicate that supplementation with rVapA increased intracellular survival of avirulent *R. equi* in a dose-dependent matter (shown in Fig. 3b). Avirulent *R. equi* supplemented with 500 nM rVapA had significantly increased intracellular survival when compared to avirulent *R. equi* without rVapA (*P* < 0.001) and did not differ from virulent *R. equi* (*P* = 0.668). Avirulent *R. equi* supplemented with rVapA at 1,000 nM had significantly higher intracellular replication compared to virulent *R. equi* (*P* < 0.001). Therefore, we used the concentration of 500 nM rVapA that mimics the replication of virulent *R. equi* in WT cells to evaluate the impact of VapA supplementation and the absence of the surface receptors TLR2, CR3, or FcγRIII in *R. equi* phagocytosis and intracellular survival in murine macrophages.

### TLR2, CR3, and FcγRIII receptors are not required for *R*. *equi* phagocytosis

**Fig 4:**
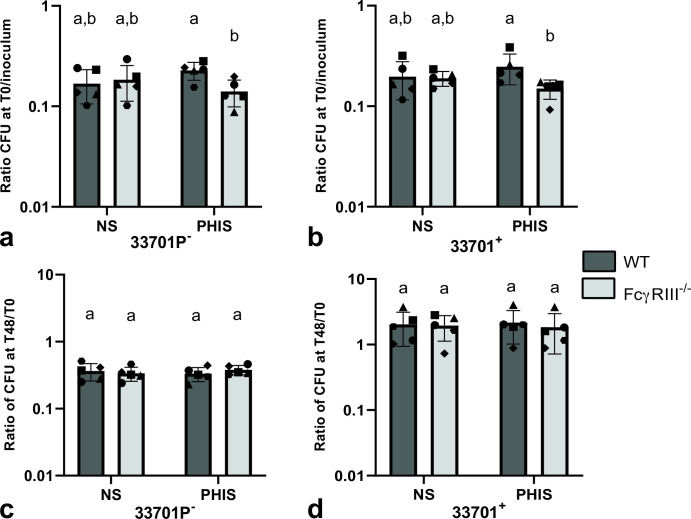
Opsonization with *Rhodococcus equi*-specific antibodies does not increase *R. equi* phagocytosis and intracellular survival in wild-type J774A.1 or FcγRIII^-/-^ compared to normal mouse serum. J774A.1 murine macrophages (WT or FcγRIII^-/-^) were infected with either virulent (33701^+^) or avirulent (33701P^-^) *R. equi* opsonized with either normal mouse serum (NS) or serum from mice hyperimmunized with β−1 → 6-linked poly-N-acetyl glucosamine (PHIS). Cells were washed and either lysed and diluted immediately (**T0**) or cultured for 48 h and then lysed (**T48**) for quantitative bacterial culture. Phagocytosis was calculated as a ratio of CFU count at T0 divided by the FU count of the bacterial inoculum. Intracellular survival was calculated as a ratio of CFU count at T48 divided by CFU count at T0. Phagocytosis (**a, b**) and intracellular killing (**c, d**) of *R. equi* opsonized with PHIS did not significantly differ from *R. equi* opsonized with NS for either virulent or avirulent *R. equi*.

Before evaluating the effects of rVapA supplementation on *R. equi* phagocytosis and intracellular survival in macrophages lacking TLR2, CR3, or FcγRIII, we demonstrated that blocking or knocking out these receptors did not diminish *R. equi* phagocytosis in either virulent or avirulent *R. equi* (shown in Fig. 2a through c). Intracellular localization of avirulent (shown in Fig. 2d; Video S1) or virulent (Fig. 1) *R. equi* was demonstrated by confocal microscopy of J774A.1 WT, TLR2^-/-^, CR3^-/-^, and FcγRIII^-/-^ infected with either GFP^+^103^+^ (virulent) or GFP^+^103^-^ (avirulent) *R. equi*. To confirm the absence of significant difference in FcγRIII^-/-^ was not due to a lack of engagement of *R. equi*-specific antibodies through this receptor, we performed opsonization with PNAG hyperimmune serum. We observed no significant difference when comparing phagocytosis of *R. equi* opsonized with either hyperimmune or normal serum (Fig. 4a and b). Interestingly, when comparing WT and FcγRIII^-/-^ opsonized with hyperimmune serum, the phagocytosis ratio was higher in WT than in FcγRIII^-/-^ for both virulent and avirulent strains (Fig. 4a and b), suggesting engagement of FcγRIII during phagocytosis of *R. equi* when specific antibodies are abundant.

### Absence of TLR2 or CR3, but not FcγRIII, increases avirulent *R. equi* intracellular replication

**Fig 5:**
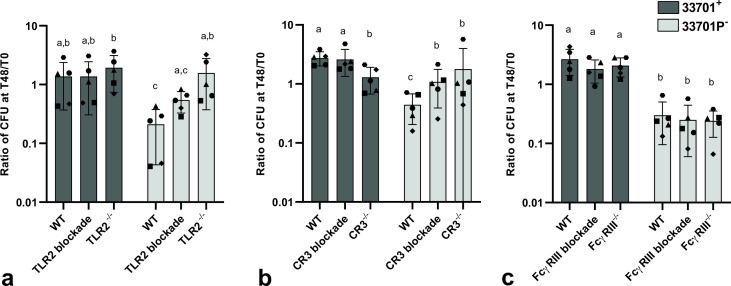
Absence of TLR2 or CR3 increases avirulent *Rhodococcus equi* intracellular replication. J774A.1 murine macrophages with surface receptors (TLR2, CR3, or FcγRIII) blocked by monoclonal antibodies (mAb) or knocked out (KO) were infected with either virulent (33701^+^) or avirulent (33701P^-^) *R. equi*. Cells were then washed and either lysed and diluted immediately (**T0**) or cultured for 48 h and then lysed (**T48**) for quantitative bacterial culture. Phagocytosis was calculated as a ratio of CFU count at T0 divided by the CFU count of the bacterial inoculum. Intracellular survival was calculated as a ratio of CFU count at T48 divided by CFU count at T0. (**a**) TLR2 blockade or KO did not affect virulent *R. equi* intracellular replication, however, avirulent *R. equi* intracellular survival was significantly increased in TLR2 KO. (**b**) CR3 mAb blockade or KO did not affect virulent *R. equi* intracellular replication; however, replication of avirulent *R. equi* in CR3 KO did not significantly differ from WT cells infected with virulent *R. equi*. (**c**) FcγRIII mAb blockade or KO did not affect intracellular survival of virulent nor avirulent *R. equi*. Five independent experiments (represented by different symbols) were performed with each experimental condition performed in triplicate. The gray bars represent the mean ratio, the error bars the standard deviation, and different letters indicate statistical difference (*P* < 0.05).

Intracellular replication of virulent and avirulent *R. equi* was significantly different in WT cells for all experiments, as expected (shown in Fig. 5a through c). TLR2, CR3, or FcγRIII blockade or KO did not interfere with intracellular replication of virulent *R. equi*. However, intracellular survival of avirulent *R. equi* significantly increased in TLR2^-/-^, while TLR2 blockade with anti-TLR2 mAb had an intermediated phenotype (shown in Fig. 5a). Intracellular replication of avirulent *R. equi* in CR3 KO macrophages was not significantly different than CR3 KO cells infected with virulent *R. equi* (shown in Fig. 5b). Knocking out or blocking FcγRIII did not affect intracellular survival of either virulent or avirulent *R. equi* opsonized with either normal (shown in Fig. 5c) or hyperimmune serum (Fig. 4c and d) .

### Absence of TLR2, CR3, or FcγRIII does not affect phagocytosis or intracellular replication of avirulent *R. equi* after rVapA supplementation

**Fig 6:**
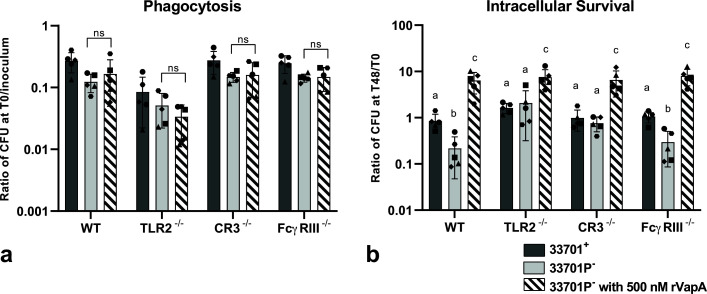
Absence of TLR2, CR3, or FcγRIII does not affect rVapA-mediated increase in *Rhodococcus equi* intracellular replication or phagocytosis. J774A.1 WT, TLR2^-/-^, CR3^-/-^, or FcγRIII^-/-^ murine macrophage were infected with either virulent (33701^+^) or avirulent (33701P^-^) *R. equi*. Before infection with avirulent *R. equi*, designated monolayers were incubated overnight with 500 nM rVapA. After infection, macrophages were then washed and either lysed and diluted immediately (**T0**) or cultured for 48 h and then lysed and diluted (**T48**) for bacterial determination. Phagocytosis was calculated as a ratio of the CFU count at T0 divided by CFU count of the bacterial inoculum. Intracellular survival was calculated as the ratio of CFU count at T48 divided by CFU count at T0. (**a**) rVapA supplementation did not alter phagocytosis of avirulent *R. equi* in WT, TLR2^-/-^, CR3^-/-^, or FcγRIII^-/-^. (**b**) rVapA (500 nM) supplementation increased avirulent *R. equi* intracellular replication (*P* < 0.001) in all cell types. Five independent experiments (represented by different symbols) were performed with each experimental condition performed in triplicate. Gray bars represent the mean ratio, the error bars the standard deviation, the letters the statistical difference (*P* < 0.05), and ns (*P* > 0.05).

**Fig 7:**
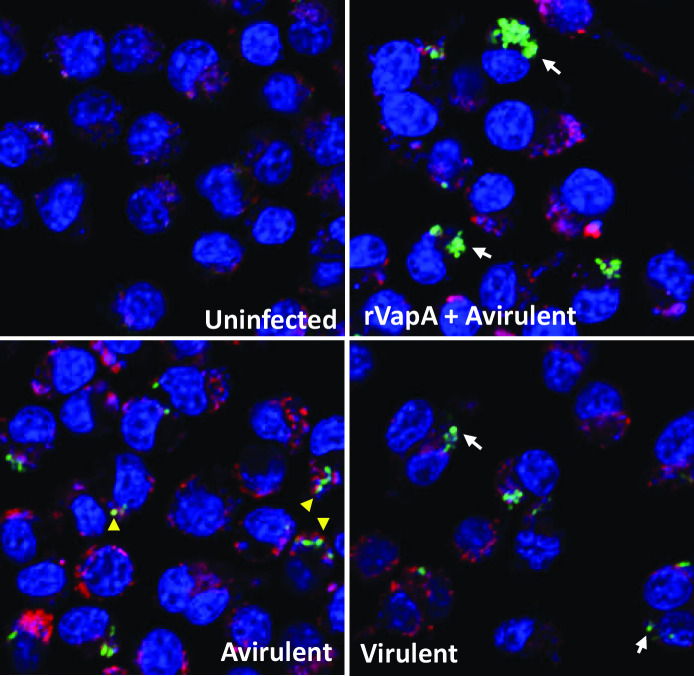
rVapA prevents acidification of macrophage infected with avirulent *R. equi*. J774A.1 murine macrophages treated or not with 500 nM of rVapA were infected with either virulent (GFP^+^103^+^) or avirulent (GFP^+^103^-^) *R. equi* and incubated for 24 h before staining. Cells were stained with Hoechst 33342 and Lysotracker Red DND-99. White arrows indicate *R. equi* without Lysotracker staining and yellow arrowhead indicate *R. equi* colocalized with Lysotracker.

**Fig 8:**
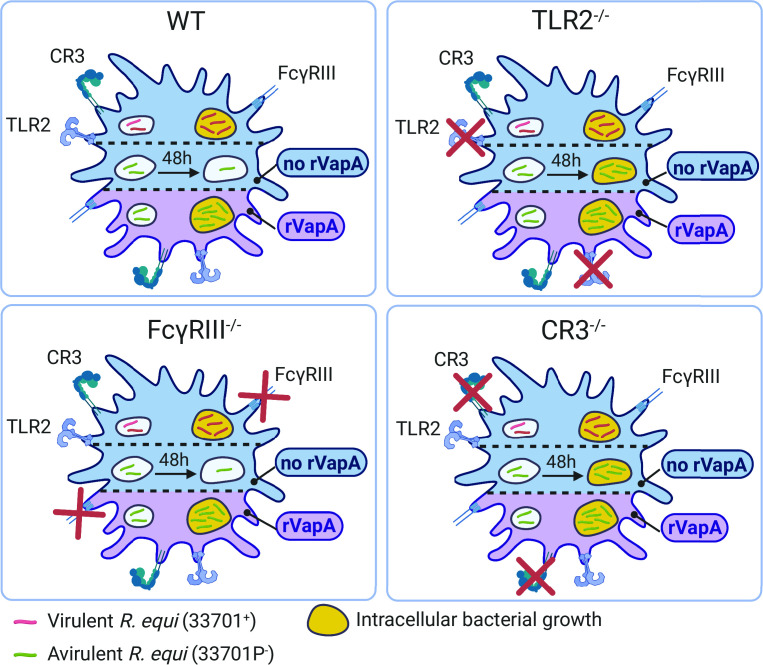
Schematic figure summarizing our results. Murine macrophages WT, TLR2^-/-^, CR3^-/-^, and FcγRIII^-/-^ were infected with either virulent or avirulent *R. equi* with (lavender) or without (blue) rVapA supplementation. In all cell types, virulent *R. equi* replicated 48 h post-infection (yellow phagolysosomes). Avirulent *R. equi* was killed by WT and FcγRIII^-/-^, but not TLR2^-/-^ and CR3^-/-^. rVapA supplementation increased replication of avirulent *R. equi* in all cell types. Created with BioRender.com.

We previously demonstrated that the absence of TLR2^-/-^, CR3^-/-^, or FcγRIII^-/-^ or rVapA supplementation did not affect phagocytosis of *R. equi* (shown in Fig. 1–3). Here, we show that combination of rVapA with the absence of receptors had no effect on phagocytosis of avirulent *R. equi* (shown in Fig. 6a). Supplementation of rVapA increased intracellular replication (*P* < 0.001) of avirulent *R. equi* (33701P^-^) in all cell types (shown in Fig. 6b), indicating rVapA supplementation alone can increase intracellular replication of avirulent *R. equi* independently of TLR2, CR3, or FcγRIII. Differences in intracellular survival of virulent and avirulent *R. equi* were abolished in TLR2^-/-^ and CR3^-/-^ macrophages, as observed previously in Fig. 5a and b. Using Lysotracker to detect lysosomal acidification in macrophages infected with *R. equi* (virulent, avirulent, or avirulent supplemented with rVapA), we observed rVapA prevented endosomal acidification of avirulent infected macrophages (Fig. 7), as previously demonstrated by other research groups (10, 52, 53). Colocalization of *R. equi* and Lysotracker was observed in cells infected with avirulent *R. equi* but not in cells infected with virulent or avirulent *R. equi* with rVapA, suggesting that rVapA prevention of acidification *of R. equi*-containing vacuoles can be a mechanism allowing avirulent *R. equi* to replicate in macrophages supplemented with rVapA (Fig. 7). Our findings are summarized in Fig. 8.

## DISCUSSION

In this study, we found that the supplementation with rVapA increases intracellular replication of plasmid-cured avirulent *R. equi* in murine macrophages in a dose-dependent manner by preventing endosomal acidification. Although similar findings have been previously described (10, 12, 13), this challenges the dogma that VapA is necessary but not sufficient for *R. equi* virulence (11). Further investigation is necessary to determine whether VapA is sufficient for virulence in equine macrophages and foals. We also evaluated whether the rVapA-associated increase in intracellular survival depended on host surface receptors previously shown to be important interaction with *R. equi* (14, 15, 37). Our data indicate that the increased intracellular replication of avirulent *R. equi* in macrophages treated with rVapA is independent of TLR2, CR3, or FcγRIII. In addition, we found that TLR2 and CR3 have a role in killing intracellular avirulent—but not virulent—*R. equi* by macrophages. Together, these data demonstrate that the mechanisms by which rVapA supplementation affects intracellular replication are independent of TLR2, CR3, or FcγRIII.

Phagocytosis of either virulent or avirulent *R. equi* was not affected by mAb blockade or knocking out TLR2, CR3, or FcγRIII receptors. The effects of blocking surface receptors with mAbs on bacterial phagocytosis vary among species. Blockade of FcγRIII or CR3 with mAbs did not affect phagocytosis of *Mycobacterium lepraemorium* (54); however, phagocytosis of *Mycobacterium tuberculosis* by macrophages was decreased following blockade of TLR2 or CR3 with mAbs (28). This variability might be explained by other factors, such as insufficient concentration of antibodies or incomplete receptor blockade depending on the binding location. Interestingly, CR3 binding was previously found to be necessary for phagocytosis of *R. equi* by macrophages (14). The authors concluded *R. equi* requires CR3 to bind to mammalian cells based on the increase in phagocytosis when opsonized with serum containing C3 in macrophages, the absence of *R. equi* binding to fibroblastoid or epithelial cells lacking CR3, and mAb CR3 blockade of murine peritoneal cells. In our study, however, we used CRISPR/Cas9 technology to generate murine macrophages with complete CR3 KO. These cells were able to phagocytose serum-opsonized *R. equi* similarly to WT cells. We demonstrated *R. equi* both intracellularly and adjacent to the cell membrane of CR3^-/-^ murine macrophages using 3D immunofluorescence images.

Intracellular survival of virulent *R. equi* in macrophages was not affected by TLR2, CR3, or FcγRIII antibody blockade or KO; however, avirulent survival increased in TLR2 and CR3 KOs. Similarly, intracellular survival of virulent *M. tuberculosis* in murine CR3^-/-^ macrophages was similar to WT (30). Also, TLR2 and CR3 blockade did not affect virulent *M. tuberculosis* intracellular survival, but it restored intracellular replication of avirulent *M. tuberculosis* (ChoD deficient) (28, 29), similar to our observations with virulent and avirulent *R. equi*. In addition, CR3^-/-^ mice infected with virulent *M. tuberculosis* did not differ in tissue infection levels or time of death compared to WT (55). TLR2^-/-^ mice have shown similar susceptibility to virulent *R. equi* infection and ability to respond to immunization (20). Together, these data suggest that TLR2 and CR3 are important in controlling the replication of avirulent *R. equi*, but the pathways downstream of these receptors fail to control virulent *R. equi* replication. Induction of the TLR2-CR3 crosstalk pathway has been reported for other bacteria, such as Mycobacteria and *Bacillus anthracis,* which might also be exploited by *R. equi*. This pathway is believed to involve bacteria inducing TLR2 inside-out signaling for transactivating and increasing CR3 binding and cellular uptake of bacteria (23–25, 56). The communication of CR3 and TLR2 is bidirectional, and CR3 can promote TIR/TIRAP/MyD88 that initiates TLR2 signaling (56, 57). Therefore, we speculate that the increased intracellular replication of avirulent *R. equi* observed in the absence of either TLR2 or CR3 might be due to the interruption of the crosstalk signaling because TLR2^-/-^ might also fail to activate CR3, or vice-versa.

Phagosomes that are formed around internalized bacteria have surface receptors such as TLR2 that contribute to sensing intracellular pathogens (58). In a study performed with *Staphylococcus aureus*, it was shown that the processes of phagocytosis of bacteria, maturation, and acidification of phagolysosomes, bacterial digestion, and release of cryptic TLR ligands inside the cell are essential to initiate optimal TLR2-MyD88-dependent response to Gram-positive bacteria (59). Activation of TLR2 by *R. equi* PAMPs controls the growth of avirulent *R. equi* by triggering bactericidal mechanisms through mitochondrial reactive oxygen species (60), NF-kB translocation (15), upregulation of pro-inflammatory genes (15), increased synthesis of lysosomal enzymes and membrane trafficking molecules (61), or other antimicrobial mechanisms, but these might be circumvented by supplementation with rVapA. Therefore, we speculate that the absence of TLR2, similar to what has been demonstrated with VapA (10, 52, 53, 62), results in failure of maturation and acidification of phagolysosomes, likely by impairment of TLR2-MyD88 pathway. The hypothesis that the absence of TLR2 impacts phagolysomes maturation and acidification following *R. equi* infection, similar to observations in other bacteria (63), needs to be further evaluated.

Here we show that the absence of FcγRIII in murine macrophages does not affect phagocytosis or intracellular survival of *R. equi* opsonized with normal mouse serum. The purported importance of this receptor originated from previous studies demonstrating that opsonization of *R. equi* either with serum or plasma increases phagocytosis and bacterial killing by phagocytes (14, 37, 39, 40), but direct evaluation of FcRs is lacking. We have previously demonstrated no effect of opsonization with either *R. equi*-HIP or standard plasma in the killing capacity of equine alveolar macrophages (39). Similarly, no significant differences were observed in clinical outcomes following *R. equi* infection from foals that previously received *R. equi*-HIP or standard plasma (64). We have also demonstrated that opsonic killing of virulent *R. equi* mediated by specific antibodies is dependent on the presence of functional complement, suggesting a limited role for FcRs in controlling *R. equi* infection (38). Here, we demonstrate that opsonization of *R. equi* with hyperimmune serum against PNAG, a polysaccharide present in the surface of both virulent and avirulent *R. equi* (44), does not significantly affect bacterial phagocytosis or intracellular survival ratios in WT nor FcγRIII^-/-^ compared to opsonization with normal mouse serum. We observed, however, a higher phagocytosis ratio by WT than FcγRIII^-/-^ macrophages when *R. equi* was opsonized with PHIS, indicating that FcγRIII engagement may be important in phagocytosis of *R. equi*. Our results, however, do not support the assumption that FcγRIII has an important role in killing intracellular *R. equi* as previously hypothesized (16). To our knowledge, this is the first study directly demonstrating that the intracellular fate of *R. equi* is independent of FcγRIII.

Our study has a number of limitations. First, our findings were not confirmed in foal macrophages. However, J774A.1 cells are phenotypically and functionally similar to equine macrophages with regard to *R. equi* infection (2, 11, 14, 65, 66). Second, the rVapA used was produced in *E. coli* and fused to GST. Despite the rVapA purification and endotoxin removal process, residual endotoxin and/or GST could have an effect on macrophage activation. However, a previous study showed no effects of either endotoxin or GST on lysosome morphology, which is associated with *R. equi* intracellular survival (67). The concentration of endotoxin in rVapA used for supplementation (0.06 EU/mL) is below acceptable endotoxin level in cell culture media (<0.25 EU/mL) and required by the United States Food and Drug Administration for medical devices and parenteral drugs (<0.5 EU/mL). In addition, despite our VapA dose-optimization experiments that indicate a concentration between 100 and 500 nM would mimic virulent *R. equi*, the dose of rVapA used in our study (500 nM) appears to have induced higher intracellular growth (Fig. 6b) and prevention of endosomal acidification (Fig. 7) in avirulent *R. equi* supplemented with rVapA compared to virulent *R. equi*. This higher dose of rVapA, however, allowed us to demonstrate that rVapA works independently of TLR2 and CR3, when avirulent *R. equi* survival was similar to virulent but increased more by rVapA. Last, our study was limited to the individual effects of surface receptors TLR2, CR3, and FcγRIII. We did not consider the combinatorial effects of receptor blockade or KO, nor did we examine other receptors such as macrophage receptors with collagenous structure (MARCO), dectin-1, or other FcRs (such as high-affinity FcγRI) that might contribute to phagocytosis or intracellular survival of *R. equi*.

Despite these limitations, we demonstrated the following important results: 1) rVapA can restore avirulent *R. equi* intracellular replication in a dose-dependent manner and prevent lysosomal acidification; 2) virulent and avirulent *R. equi* can be in internalized by murine macrophages in the absence of TLR2, CR3, or FcγRIII similarly to WT; 3) TLR2^-/-^ or CR3^-/-^ macrophages fail to control intracellular replication of avirulent *R. equi*; and, 4) the capacity of rVapA to promote intracellular replication of avirulent *R. equi* does not require TLR2, CR3, or FcγRIII surface receptors. Our results indicate TLR2 and CR3 contribute to control the intracellular replication of avirulent *R. equi* but not virulent *R. equi*, and that the mechanisms of action of VapA are independent of these receptors.

## SUPPLEMENTAL MATERIAL

Supplemental figures and video legend iai.00383-23-s0001.docx Fig. S1 to S4 and legend of Video S1.

Supplemental movie iai.00383-23-s0002.mp4 Video S1, legend included in the supplemental material file.

ASM does not own the copyrights to Supplemental Material that may be linked to, or accessed through, an article. The authors have granted ASM a non-exclusive, world-wide license to publish the Supplemental Material files. Please contact the corresponding author directly for reuse.
