# Supplementary material for: Impact of surface receptors TLR2, CR3, and FcγRIII on Rhodococcus equi phagocytosis and intracellular survival in macrophages
Source: Infect Immun. 2023 Nov 29;92(1):e00383-23. doi: 10.1128/iai.00383-23 (PMC10790823; doi:10.1128/iai.00383-23)
Supplement: Supplemental figures and video legend — Fig. S1 to S4 and legend of Video S1. [file iai.00383-23-s0001.docx]

**Supplementary Material**

**
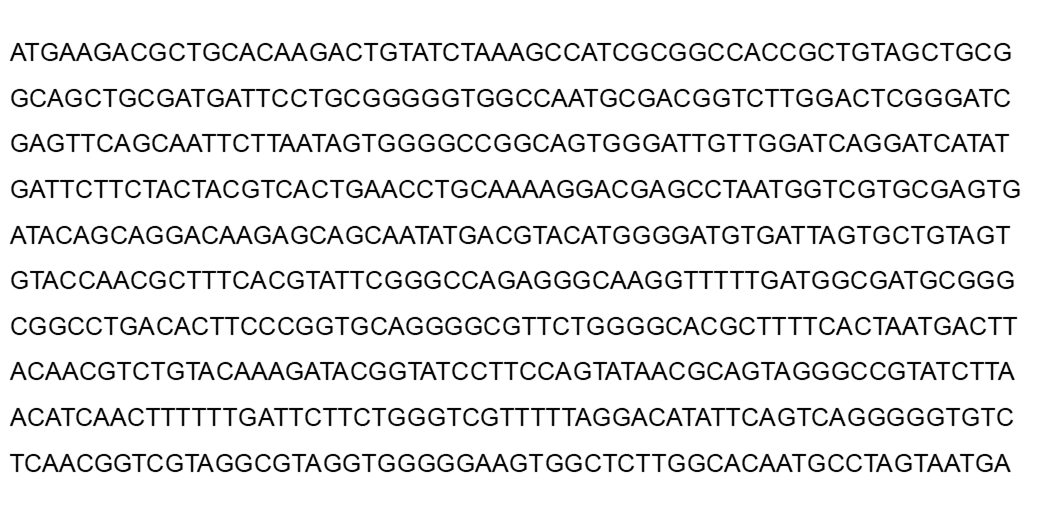
Figure S1: DNA sequence vapA codon optimized for *E. coli* expression**


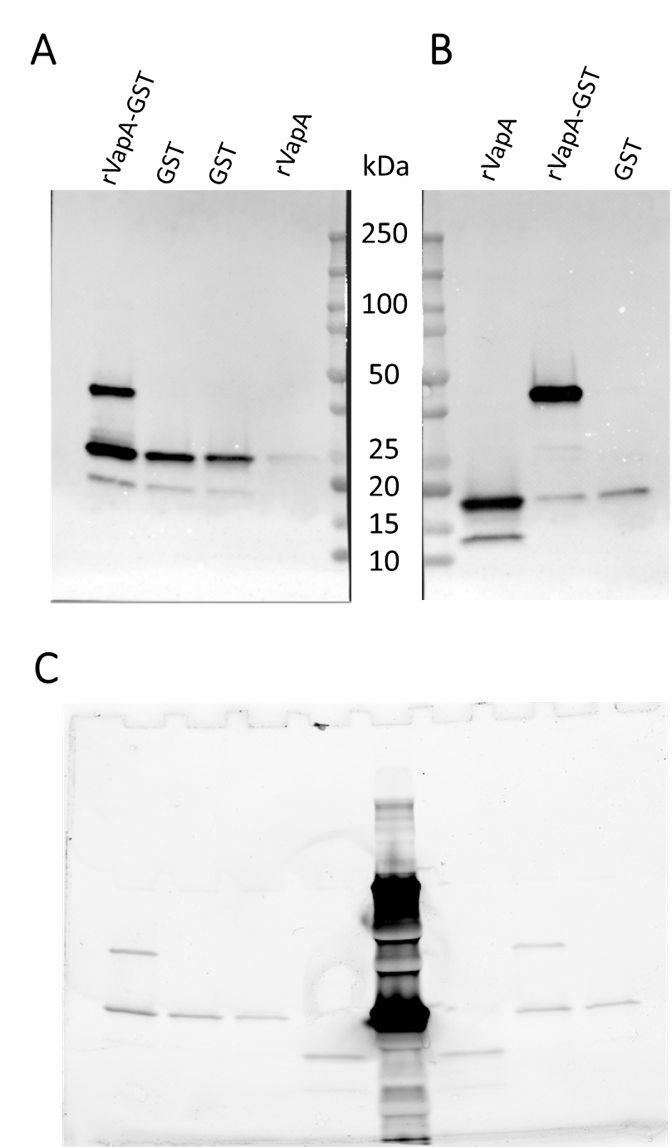


**Figure S2**. rVapA purification. **(A)** Western blot of GST-rVapA protein including samples before (GST-rVapA) and after GST removal (GST fraction and rVapA fraction) stained with anti-GST antibody (Supplementary Table S2). **(B)**. Western blot of GST-rVapA protein including samples before (GST-rVapA) and after GST removal (GST fraction and rVapA fraction) stained with anti-VapA antibody (Supplementary Table S2). **(C)**. Total protein gel before membrane transfer and western blot were performed (A & B).

**
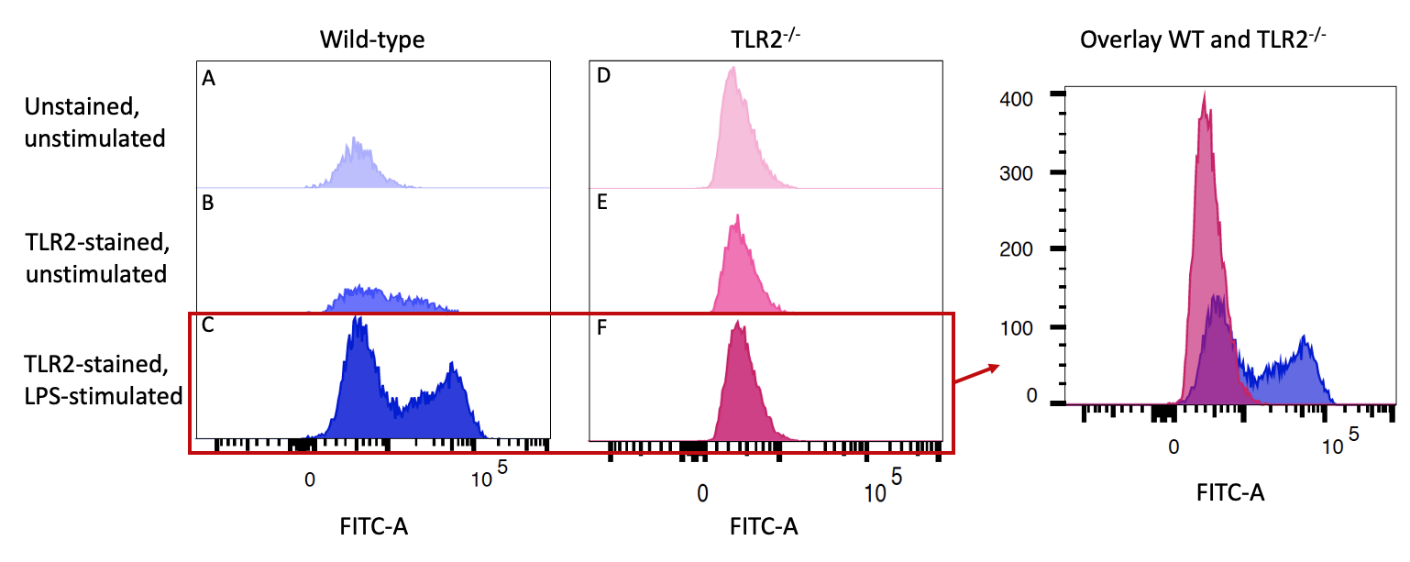
Figure S3**. Flow cytometry of J774A.1 WT and TLR2^-/-^ stained with anti-mouse TLR2-FITC antibody, and stimulated with either 0 or 500 ng/m LPS or media for 8 hours to induce expression of TLR2. Left panel: A) unstained and unstimulated WT J774A.1 cells; B) anti-TLR2 stained and unstimulated WT J774A.1 cells; C) anti-TLR2 stained and LPS-stimulated WT J774A.1 cells. Middle panel: D) unstained and unstimulated TLR2^-/-^ J774A.1 cells; E) anti-TLR2 stained and unstimulated TLR2^-/-^ J774A.1 cells; F) anti-TLR2 stained and LPS-stimulated TLR2^-/-^ J774A.1 cells. Right panel: overlay of stained and stimulated cells (blue – WT; pink – TLR2^-/-^).

**Figure S4**. J774A.1 murine macrophage monolayers were infected with either fresh or frozen cultures of virulent (33701^+^) or avirulent (33701P^-^) *R. equi*. Macrophages were then washed and either lysed and diluted immediately for bacterial determination (T0), or cultured for 48 h and then lysed and diluted (T48) for bacterial determination. Phagocytosis was calculated as a ratio of CFU count at T0 divided by CFU count of the bacterial inoculum. Intracellular survival was calculated as a ratio of CFU count at T48 divided by CFU count at T0. **(A)** No significant differences in the phagocytosis ratio between fresh cultured or frozen bacterial inoculum of virulent and avirulent *R. equi*. **(B)** Intracellular survival was significantly higher for virulent *R. equi* than avirulent, however, there was no significant difference between fresh or frozen bacterial inoculum. Three independent experiments (represented by different symbols) were performed with each experimental condition performed in triplicate. The gray bars represent the mean ratio, the error bars the standard deviation, and ns represent no significant statistical difference (P > 0.05) between tested conditions.

**Video S1**: J774A.1 WT cells were infected with avirulent (GFP^+^103^-^) *R. equi* (green). Immunostaining of CR3 (CD11b-AF647; red) and nuclei stained with Hoechst 33342 (blue) were performed.
